# Psychosis Risk and Generative Artificial Intelligence Use Frequency, Motivations, and Delusion-Like Experiences: Cross-Sectional Survey Study

**DOI:** 10.2196/85038

**Published:** 2026-03-05

**Authors:** Benjamin Buck, Anne Julia Maheux

**Affiliations:** 1 Department of Psychiatry University of North Carolina at Chapel Hill Chapel Hill, NC United States; 2 Department of Psychology and Neuroscience University of North Carolina at Chapel Hill Chapel Hill, NC United States

**Keywords:** digital health, digital mental health, artificial intelligence, generative AI, psychosis risk, psychosis, young adults, mental health

## Abstract

**Background:**

Growth of generative artificial intelligence (GenAI) has exploded in recent years. Many have noted its substantial potential to increase access to scalable digital mental health interventions or provide companions for individuals who are socially isolated. At the same time, seeking mental health support from mainstream GenAI models may involve risks. Several recent examples of exacerbation of delusions have received attention in the popular press, leading to a call for empirical research to document the scope of interactions with GenAI among individuals experiencing symptoms of psychosis.

**Objective:**

This study aimed to evaluate associations of psychosis risk to GenAI use frequency, motivations for use, and GenAI interactions involving potential delusions.

**Methods:**

We conducted a large-scale cross-sectional survey of 1003 young adults in United States, divided the sample of individuals that had used GenAI into “elevated risk” (Prodromal Questionnaire, Brief Version Distress Score ≥20; N=267, 28%) and “low risk” groups (Prodromal Questionnaire, Brief Version Distress Score <20; N=685, 72%), and compared groups on several assessments related to GenAI use.

**Results:**

We found that while members of the elevated risk group were no more likely to have ever used GenAI, they were significantly more likely to report intensive use (odds ratio 1.70 to 2.56; ie, several times per day, more than 30 minutes per day, 6 or more chatbot conversations per day). Those at elevated risk were more likely to report using GenAI to receive social and emotional support and significantly more likely to ascribe human-like roles to their chatbot interactions (odds ratio 1.76 to 3.08; ie, companion, friend, therapist, and romantic partner). Delusion-related interactions were also commonly reported among those at risk for psychosis (item endorsements from 13.3% to 30.7%).

**Conclusions:**

While it is unclear whether they have a positive or negative impact overall, GenAI chatbots may have the potential to impact symptom-related experiences among young adults at risk.

## Introduction

Generative artificial intelligence (GenAI) is now the most rapidly adopted technology in history [1]. In July 2025, less than 3 years after its launch in November 2022, ChatGPT (OpenAI) boasted 700 million users, or roughly one tenth of the world’s population [2]. Beyond ChatGPT, several others have experienced similar growth, including other general-use artificial intelligence (AI) platforms (eg, Claude [Anthropic] and Gemini [Google]), those focused on image and video generation (eg, Stable Diffusion), and others that allow for interaction with customizable characters (eg, Character.AI). Many have noted the potential of GenAI technologies to deliver scalable and self-guided mental health interventions [3], with emerging evidence demonstrating efficacy of interventions leveraging GenAI relative to waitlist control [4]. Survey studies suggest that GenAI systems are already used both by individuals seeking mental health support and by mental health providers to increase efficiency (eg, documenting clinical interactions or researching clinical literature) [5], and prevalence of use is nearly certain to increase in years to come.

While the potential benefit in expanding access to mental health interventions and resources is clear, many have also been monitoring for potential risks, particularly when unrestricted, general use chatbots are used for mental health support [6]. One particular concern is sycophantic behavior, wherein GenAI chatbots overly flatter or agree with users [7], which has been noted in general use GenAI [8]. Another is hallucination or misinformation, a widely observed phenomenon wherein GenAI chatbots provide incorrect details with an overall convincing presentation [9]. Some of these limitations may strengthen atypical or pathological beliefs rather than challenge them. One prominent example of harm occurred in 2023, when Tessa, a chatbot provided by the National Eating Disorders Association, provided problematic guidance related to eating disorders, including encouraging strategies for weight loss [10]. While more recent research on GenAI chatbots for mental health have demonstrated improved efficacy and capacity for preventing negative outcomes [4,11], there remain questions about risks associated with GenAI use for mental health concerns, particularly when the GenAI platforms are not specialty systems designed for such a purpose and with guardrails.

An emerging concern in this area is psychosis risk [12]. Psychosis symptoms can be arranged along a transdiagnostic risk continuum, ranging from common and low severity symptoms (eg, fleeting and infrequent hallucinatory experiences, preoccupation with odd beliefs) to rare, highly severe symptoms (eg, ongoing command hallucinations, highly distressing and high-conviction delusions) that are often linked with negative outcomes [13]. Schizophrenia-spectrum disorders emerge following a prodromal period (typically occurring in late teens to mid-twenties) wherein individuals experience subthreshold symptoms and changes from premorbid functioning. Experiencing these prodromal symptoms is associated with a significant increase in likelihood of both schizophrenia-spectrum [13] or other psychiatric disorders [14]. Young people within the age group that is at the highest risk for first developing a psychotic disorder are among the highest adopters of GenAI [1]. Individuals at elevated risk for psychosis are also more likely to engage in problematic use of other digital media [15-17], and such problematic use appears associated with elevated stress in this population [18,19] as well as risk for future symptoms [20]. Taken together, these findings suggest a need for research to understand the use of GenAI among individuals at elevated risk for psychosis.

Several well-publicized examples of troubling interactions with GenAI systems have raised concerns about the ways these technologies could introduce new risks for individuals at risk for psychosis. In several interactions described in the popular press, users have shared delusions or odd beliefs with the model, and in response, the GenAI chatbot has reportedly responded in ways that seem to have increased delusional conviction, distress, or behavioral responses. For example, in one case, a 29-year-old mother of 2 came to believe that ChatGPT could facilitate “interdimensional communication” [21]. In another, a 35-year-old man with a history of bipolar disorder and schizophrenia became fixated on an AI entity called Juliet; when he came to believe that OpenAI executives had killed this entity, he threatened to kill them and was killed after attacking law enforcement with a knife [21]. In other, less tragic examples, individuals with no history of mental illness have embraced grandiose narratives about their own knowledge or abilities [22]. While these extreme examples are a concern, there is a lack of evidence to inform the public as to the prevalence of these risks at the population level. It is unclear the extent to which publicized cases with negative outcomes may indicate a more common phenomenon. A first step in answering this question involves assessing the amount and type of GenAI use among young adults at varying risk levels for psychosis, evaluating whether their interactions with GenAI chatbots differ in meaningful ways from the general population, and to assess the frequency of interactions that could impact mental health outcomes. There is a lack of empirical studies on relationships of psychosis risk to (1) GenAI use frequency and (2) motivations for GenAI use, and (3) delusion-related interactions with GenAI models.

Our team conducted a large-scale (N=1003) survey of young adults in July 2025. In addition to measures of GenAI use frequency, motivations for GenAI use, and GenAI experiences, we collected a screening questionnaire assessing psychosis risk. In this study, we examine cross-sectional relationships of GenAI variables to symptoms indicating elevated psychosis risk. Based on literature suggesting elevated rates of problematic use of digital media among individuals at risk for psychosis, we expect that individuals at elevated risk will be more likely to report the highest patterns of use and to use GenAI for social and emotional support. We also expect that individuals at elevated risk will be more likely to report interacting with GenAI about symptom-related content. Study results could provide an important first step to identifying the potential risks and benefits of these platforms for individuals at risk of developing psychosis, as well as characterizing their scale and level of impact.

## Methods

### Study Design

We collected a cross-sectional survey in July 2025 via the online crowdsourcing platform Prolific. Our survey was open to all Prolific users, given they met our inclusion criteria of (1) residing in the United States and (2) being between ages 18 and 25 years. Convenience sampling was used as our sample comprised all individuals who responded to the study listing. Our study had multiple goals. First, we aimed to examine overall relationships between psychosis risk and self-reported use of GenAI (eg, lifetime use, frequency, and average duration of use). Second, we sought to characterize motivations for and types of GenAI use by examining associations with psychosis risk symptoms. Third, we developed a measure to assess delusion-related interactions with GenAI systems to characterize the frequency with which individuals at elevated risk for psychosis reported these experiences.

### Participants

Participants were young adults between the ages of 18 and 25 years in the United States (for sample demographics, see Multimedia Appendix 1). On Prolific, participants can complete screening, provide identity verification, and receive compensation through Prolific without sharing identifying information with researchers. Thus, all study participants were anonymous to the study team, and all received information about crisis support or finding mental health resources during study participation. This sample includes all individuals who provided sufficient responses to the Prodromal Questionnaire, Brief Version (PQ-B [23]) to generate an estimate of psychosis risk (ie, 14 or more of the 21 items). This exclusion criterion resulted in removing only 7 participants from the original study sample. In addition to 44 (4.4%) that were removed for missing at least 1 of 2 attention checks, this resulted in a final sample of 952 participants.

### Measures

Psychosis risk was assessed with the PQ-B [23]. The PQ-B is widely used in identifying individuals experiencing symptoms that may indicate risk for psychotic episodes (ie, prodromal symptoms). The PQ-B has 21 items that are rated on their occurrence and a Likert scale assessing distress related to items that the respondent endorsed. While a high score on the PQ-B does not on its own indicate meeting clinical high-risk criteria (a gold-standard determination of this is made by a clinician), it does indicate that a formal evaluation is more likely to find that the respondent is indeed at risk. And in population-level research, the PQ-B Distress Score, which captures both symptoms endorsed and associated distress, can be used continuously as a measure of psychosis risk symptoms. Systematic reviews of the PQ-B have identified optimal cutoffs for screening and referral for further evaluation in both clinical and nonclinical settings [24]. For analyses that require identifying a subset of participants at elevated risk, we follow a provisional cutoff of 20 used in prior work in both clinical [25] and online settings [26] to generate “elevated risk” (PQ-B Distress Total ≥20) and “low risk” (PQ-B Distress Total <20).

Our team developed measures to evaluate AI use frequency, motivations for use, and interactions with GenAI involving delusion-like experiences. Regarding use, we administered 4 items addressing frequency, recency, typical length of sessions, and typical number of daily sessions (see Multimedia Appendix 2 for items and response options). The Artificial Intelligence Motivation and Uses Scale (AIMUS) surveys participants’ motivations for and types of use of GenAI systems in the following categories, derived via a factor analysis conducted by our team that is as yet unpublished [27] (see Multimedia Appendix 1 for more psychometric information about the scale, all items, and factor loadings): (1) task automation (ie, completing onerous or rote tasks with the assistance of the system), (2) learning and exploration (ie, searching for information, generating ideas, or receiving help understanding difficult topics), (3) emotional support (ie, support in managing emotions, thinking through decisions, or managing worrying thoughts), (4) dating and sexuality (ie, interacting with GenAI in romantic or sexual role-plays). Participants endorse items on a Likert scale ranging from 1 (“Never”) to 5 (“Very Often”). Subscale scores were mean imputed for individuals that completed at least half the items on the scale. We also asked one item about relationships that respondents felt they had with AI systems. For these items, participants were asked to report whether they considered any AI system to be a companion, a therapist, a friend, a romantic partner, or a sexual partner.

Delusion-like experiences involving AI were assessed with the Generative AI Aberrant Thoughts and Experiences Scale (GAATES). To develop the GAATES, study team members identified the most common delusion categories from recent meta-analytic work on psychosis [28], and created items specific to GenAI interactions. These depicted both (1) potential delusions about AI (eg, paranoid thoughts about AI, grandiose thoughts about one’s interactions with AI), as well as (2) uses of AI that may maintain existing delusions (eg, interacting with AI to reinforce one’s paranoid or grandiose beliefs). Item responses range from 1 (“Strongly Disagree”) to 5 (“Strongly Agree”). Exploratory factor analysis indicated that the scale is unidimensional, with all items loading (loadings >.45) onto a single factor that explained 53% of the variance in the underlying construct. For analyses examining endorsement of each phenomenon as a binary variable, responses of “Agree” or “Strongly Agree” (ie, 4 or 5) were coded as endorsement, while all other responses (including 3, “Neither Agree nor Disagree”) were coded as nonendorsement. Scale internal consistency was high (Cronbach α=0.95). Full item responses by group as well as results from the psychometric analyses (eg, factor analysis) are reported in the Multimedia Appendix 1.

### Data Analysis

Given the nature of the scientific question, we took an exploratory approach to characterize relationships and group differences broadly across a variety of constructs. Aside from the variable related to previous GenAI use itself, all analyses included only the subset of the sample reporting current or previous GenAI use. Alpha level for statistical significance was set at .05. First, to examine the relationships of psychosis risk to use frequency variables, we split the sample based on frequency responses indicating intensive use of GenAI. This generated binary variables representing four comparisons: (1) individuals who reported using GenAI several times per day versus less, (2) those who reported using GenAI the day of the survey versus longer ago, (3) those who reported using GenAI more than 30 minutes per session versus shorter than this, and (4) those who reported having 6 or more conversations with the chatbot per day versus 5 or fewer. We conducted chi-square tests and computed odds ratios (ORs) based on the cross product of cells in contingency tables, with CIs based on the standard error of the log ORs. PQ-B Distress scores across were compared across high versus low GenAI frequency using independent samples *t* tests and as well as chi-square tests and ORs (also derived from contingency tables) to characterize the frequency of intensive AI use among each psychosis risk group (elevated risk vs low risk). Cohen *d* estimates were calculated using pooled SDs. Second, to evaluate relationships to AI motivations, we examined Pearson correlations of PQ-B Distress scores to each subscale on the AIMUS. Third, to assess relationship variables, we compared the frequency of endorsing each relationship (friend, therapist, companion, romantic partner, and sexual partner) among each risk group using chi-square tests compared PQ-B Distress scores among individuals who endorsed each relationship using independent samples *t* tests. Finally, we examined the Pearson correlation of the GAATES to PQ-B Distress score and compared frequency of endorsing each delusion-related item across risk groups using chi-square tests and ORs. For all Pearson correlations, CIs were generated using Fisher *r*-to-*z* transformations. All linear models were evaluated for assumption violations; nonnormal variables were reanalyzed with appropriate nonparametric models and/or variable transformations, and the pattern of results were unchanged (Multimedia Appendix 1).

### Ethical Considerations

All research procedures were reviewed by the institutional review board at the University of North Carolina at Chapel Hill and were deemed exempt (IRB#25-1686) on June 26, 2025. All participants provided informed consent through online data capture, and because no individually identifying information was collected, data were anonymized. Based on an estimated 30-minute completion time and an intended compensation rate of US $8 per hour, participants were compensated US $4 for their time.

## Results

### Frequency and Average Length of Use

Of the sample of 952, 846 (88.9%) had used GenAI, while the remainder (N=106, 11.1%) had not. There were no significant differences in the PQ-B Distress score between individuals who had never used GenAI platforms (mean 13.05, SD 17.06) and those who had (mean 13.90, SD 17.14; mean difference=–0.85, 95% CI –4.31 to 2.61; *t*_950_=–0.48; Cohen *d*=–0.05, 95% CI –0.25 to 0.15; *P*=.63). Participant demographics can be found in Multimedia Appendix 1.

Regarding frequency, individuals who reported using GenAI “several times per day” or more had significantly elevated PQ-B Distress scores (113/845, 13.4%; mean 21.75, SD 20.98) relative those reporting using GenAI less frequently (732/845, 86.6%; mean 12.71, SD 16.15), mean difference=–9.04 (95% CI –12.39 to –5.69), *t*_843_=–5.30, *P<*.001, Cohen *d*=–0.54 (95% CI –0.74 to –0.34). Individuals who reported that their most recent use of GenAI was the day of the survey (ie, “Today”; N=287, 34.0%) had significantly elevated PQ-B Distress scores (mean 17.00, SD 19.54) relative to those who reported that their most recent use was the day prior or earlier (558/845, 66.0%; mean 12.33, SD 15.54; mean difference=–4.66, 95% CI –7.09 to –2.24; *t*_843_=–3.77; Cohen *d*=–0.27, 95% CI –0.42 to –0.13; *P*<.001). Individuals that reported that they would initiate over 5 conversation sessions per day (96/803, 12.0%) also had significantly elevated PQ-B Distress scores (mean 20.51, SD 21.42) relative to those who reported having 5 conversation sessions or fewer per day (707/803, 88.0%; mean 13.15, SD 16.53; mean difference=–7.36, 95% CI –11.03 to –3.69; *t*_801_=–3.94; Cohen *d*=–0.43, 95% CI –0.64 to –0.21; *P<*.001). Expressed as ORs in binary analyses (Table 1), individuals at elevated risk for psychosis (using PQ-B Distress Total ≥ 20) had more than double the odds of being in the respective intensive use category regarding frequency of use and number of conversation sessions, and they were over 50% higher odds of reporting GenAI use on the day of the survey. There were no differences between groups divided on the variable of length of each episode of use.

**Table 1:** Responses to generative artificial intelligence frequency items among individuals endorsing lifetime generative artificial intelligence use (N=846). Numbers vary by item as a result of missing responses.

Variables			Low risk (n=605), n (%)		Elevated risk (n=241), n (%)		OR^a^ (95% CI)			Chi-square (*df*)			*P* value	
How often do you use generative AI^b^ chatbots (eg, ChatGPT, Claude, and Gemini) for any purpose? (N=845)								2.56 (1.71-3.83)			21.62 (1)			<.001
	Less than “Several Times Per Day” (n=732, 86.6%)	544 (90.1)		188 (78.0)										
	“Several Times Per Day” (n=113, 13.4%)	60 (9.9)		53 (22.0)										
When was the last time you used a generative AI chatbot? (N=845)								1.70 (1.25-2.32)			11.57 (1)			<.001
	Any time before “Today” (n=558, 66%)	420 (69.5)		138 (57.3)										
	“Today” (n=287, 34%)	184 (30.5)		103 (42.7)										
When you do use generative AI chatbots, how much time do you typically spend per session? (N=846)								1.32 (0.84-2.08)			1.42 (1)			0.23
	Less than 30 minutes (n=751, 88.8%)	542 (89.6)		209 (86.7)										
	More than 30 minutes (n=95, 11.2%)	63 (10.4)		32 (13.3)										
On a day when you use a generative AI chatbot, how many separate times do you typically start a new conversation or ask it about something different? (N=803)^c^								2.10 (1.36-3.25)			11.49 (1)			<.001
	Five or fewer times (707, 88%)	516 (90.5)		191 (82.0)										
	Six or more times (96, 12%)	54 (9.5)		42 (18.0)										

^a^OR: odds ratio.

^b^AI: artificial intelligence.

^c^This excludes responses of “I don’t know/I haven’t paid attention,” which were provided as a response item for this question.

### Motivations for AI Use

All motivations for use were positively associated with psychosis risk in the full sample, with the strongest correlations being between PQ-B distress scores and emotional support motivations (*r*=0.33, 95% CI 0.26-0.38; *P<*.001), followed by dating and sexuality (*r*=0.23, 95% CI 0.16-0.29; *P<*.001), task automation (*r*=0.15, 95% CI 0.08-0.21; *P<*.001) and learning and exploration (*r*=0.14, 95% CI 0.08 to 0.21; *P<*.001).

### AI Relationship Variables

Of the AI relationships individuals reported, items ranged in endorsement from around 3% (AI as a sexual partner; 26/846, 3.1%) to over a third (AI as a companion; 315/846, 37.2%) of all participants in the sample who had previously used GenAI. PQ-B Distress Scores were higher for individuals that endorsed feeling that AI served as a companion (mean 16.75, SD 18.73 vs mean 12.20, SD 15.89; mean difference=–4.55, 95% CI –6.92 to –2.18; *t*_844_=–3.76; Cohen *d*=–0.27, 95% CI –0.41 to –0.13; *P<*.001), therapist (mean 20.77, SD 20.59 vs mean 11.10, SD 14.63; mean difference=–9.67, 95% CI –12.14 to –7.21; *t*_844_=–7.70; Cohen *d*=–0.58, 95% CI –0.74 to –0.43; *P<*.001), friend (mean 18.70, SD 19.53 vs mean 11.30, SD 15.08; mean difference=–7.40, 95% CI –9.77 to –5.03; *t*_844_=–6.12; Cohen *d*=–0.44, 95% CI –0.58 to –0.30; *P<*.001), and romantic partner (mean 21.97, SD 18.89 vs mean 13.58, SD 17.00; mean difference=–8.39, 95% CI –14.43 to –2.35; *t*_844_*=*–2.73; Cohen *d*=–0.49, 95% CI –0.85 to –0.14; *P*=.007). Examined as odds ratios between risk groups (Table 2), GenAI users in the elevated risk group had approximately threefold higher odds of reporting seeing GenAI as a therapist, more than double the odds of seeing GenAI as a friend or romantic partner, and more than 70% higher odds of seeing GenAI as a companion. There were no differences between the groups in the item related to viewing GenAI as a sexual partner.

**Table 2:** Responses to generative artificial intelligence relationship items among individuals endorsing lifetime generative artificial intelligence use (N=846). Numbers vary by item as a result of missing responses.

Variables			Low risk (n=605), n (%)		Elevated risk (n=241), n (%)		OR^a^ (95% CI)			Chi-square (*df*)			*P* value	
Companion (N=846)								1.76 (1.30-2.39)			13.44 (1)			<.001
	No (n=531, 62.8%)	403 (66.6)		128 (53.1)										
	Yes (n=315, 37.2%)	202 (33.4)		113 (46.9)										
Therapist (N=846)								3.08 (2.24-4.24)			50.24 (1)			<.001
	No (n= 601, 71%)	472 (78.0)		129 (53.5)										
	Yes (n=245, 29%)	133 (22.0)		112 (46.5)										
Friend (N=846)								2.52 (1.85-3.43)			35.61 (1)			<.001
	No (n=549, 64.9%)	430 (71.1)		119 (49.4)										
	Yes (n=297, 35.1%)	175 (28.9)		122 (50.6)										
Romantic partner (N=846)								2.62 (1.29-5.32)			7.56 (1)			.006
	No (n=814, 96.2%)	589 (97.4)		225 (93.4)										
	Yes (n=32, 3.8%)	16 (2.6)		16 (6.6)										
Sexual partner (N=846)								0.92 (0.38-2.22)			0.03 (1)			.86
	No (n=820, 96.9%)	586 (96.9)		234 (97.1)										
	Yes (n=26, 3.1%)	19 (3.1)		7 (2.9)										

^a^OR: odds ratio.

### Delusion-Like Experiences

GAATES scores differed between individuals at elevated risk (mean 36.22, SD 14.74) and those at low risk (mean 26.39, SD 10.33; mean difference=–9.83, 95% CI –11.59 to –8.07; *t*_844_=–10.98; Cohen *d*=–0.84, 95% CI –0.99 to –0.68; *P<*.001). Total GAATES scores significantly correlated with PQ-B Distress in the full sample (*r*=0.40, 95% CI 0.34-0.45; *P<*.001) as well as separately in the elevated risk group (*r*=0.28, 95% CI 0.16-0.39; *P<*.001) and the low risk group (*r*=0.11, 95% CI 0.03-0.19; *P*=.008). For all items, rates of endorsement frequency differed between risk groups (Table 3). Several of the items that most differed between those at low and elevated risk most often pertained to paranoia or persecutory ideation (eg, “AI provides me facts about how others are working to harm me”; 41/241, 17% in the elevated risk group vs 19/605, 3.1% in the low-risk group; “AI is being used by others to harm me”, 33/241, 13.7% vs 17/605, 2.8%; “AI has shown me how others are trying to control my actions”, 61/241, 25.3% vs 37/605, 6.1%), while others related to grandiosity (“I’ve discovered hidden or secret truths about the world through AI”, 43/241, 17.8% vs 22/605, 3.6%; “AI helps me make sense of secret messages (eg, from television or the news) that were intended only for me”, 58/241, 24.1% vs 42/605, 6.9%) also clearly distinguished the groups.

**Table 3:** Responses to individual Generative AI Aberrant Thoughts and Experiences Scale items among individuals endorsing lifetime generative artificial intelligence use (N=846).

Variables	Low risk (n=605), n (%)	Elevated risk (n=241), n (%)	OR^a^ (95% CI)	Chi-square (*df*)	*P* value
AI^b^ tries to read or manipulate my thoughts	36 (6.0)	38 (15.8)	2.96 (1.83-4.80)	20.81 (1)	<.001
AI tries to control my behavior	25 (4.1)	33 (13.7)	3.68 (2.14-6.34)	24.67 (1)	<.001
AI helps me understand that others are reading or manipulating my thoughts	51 (8.4)	57 (23.7)	3.37 (2.23-5.09)	35.86 (1)	<.001
AI has shown me how others are trying to control my actions	37 (6.1)	61 (25.3)	5.20 (3.35-8.09)	62.00 (1)	<.001
AI communicates things to me that only I can understand	46 (7.6)	61 (25.3)	4.12 (2.71-6.26)	48.92 (1)	<.001
AI can reveal the truth that I am a special, unique, or powerful person	53 (8.8)	68 (28.2)	4.09 (2.75-6.09)	53.22 (1)	<.001
AI helps me make sense of secret messages (eg, from television or the news) that were intended only for me	42 (6.9)	58 (24.1)	4.25 (2.76-6.54)	48.49 (1)	<.001
AI interacts with me in a special way because of who I am	46 (7.6)	62 (25.7)	4.21 (2.77-6.39)	50.83 (1)	<.001
AI is being used to secretly monitor me specifically	28 (4.6)	33 (13.7)	3.27 (1.93-5.54)	21.17 (1)	<.001
AI is being used by others to harm me	17 (2.8)	33 (13.7)	5.49 (2.99-10.06)	36.71 (1)	<.001
AI helps me learn how people are spying on or monitoring me	20 (3.3)	32 (13.3)	4.48 (2.51-8.00)	29.71 (1)	<.001
AI provides me facts about how others are working to harm me	19 (3.1)	41 (17.0)	6.32 (3.59-11.15)	50.33 (1)	<.001
AI systems are at their core an attempt by powerful people to control the world	89 (14.7)	74 (30.7)	2.57 (1.80-3.66)	28.35 (1)	<.001
AI systems use data from their users to influence world events	64 (10.6)	65 (27.0)	3.12 (2.13-4.59)	35.84 (1)	<.001
I’ve discovered hidden or secret truths about the world through AI	22 (3.6)	43 (17.8)	5.76 (3.36-9.86)	49.04 (1)	<.001
I have gained access to information through AI about the true nature of the world that I could not find in mainstream sources	43 (7.1)	56 (23.2)	3.96 (2.57-6.09)	43.39 (1)	<.001

^a^OR: odds ratio.

**^b^**AI: artificial intelligence.

## Discussion

### Principal Findings

Due to a growing collection of stories publicized by the popular press, interest has grown regarding the mental health impacts of GenAI systems among individuals prone to psychosis. This study is the first of its kind, to our knowledge, to examine the cross-sectional relationships of psychosis risk to GenAI use, motivations, and delusion-related interactions. Results suggest that while individuals at elevated risk for psychosis do not differ from the general population in likelihood to have ever used GenAI, they may indeed be more likely to use these systems at high levels of frequency, to seek emotional support from them, and to engage in interactions related to preexisting aberrant beliefs, including paranoia, grandiosity, and conspiracy thinking. While it remains unclear at present whether these interactions may on balance affect the mental health of this population in a positive or negative manner, our results suggest that they are widely used and thus, those impacts—whatever they may be—may be occurring at a large scale.

First, regarding frequency of use, psychosis risk appeared linked with likelihood to engage in the highest levels of GenAI use. Individuals at elevated risk for psychosis had more than twice the odds of using GenAI several times per day or having six or more initiated GenAI conversations per day as those at low risk. They were also significantly more likely to report having last used GenAI the same day that they took the survey. These findings align with extant research that suggests that individuals at elevated risk for psychosis are more likely to engage in intensive internet use [15,29,30]. Studies demonstrating a link of psychosis risk to media use focus on extreme or problematic use, while other studies examining more continuous relationships have found more mixed results [20,31,32]. Analogous research on GenAI is lacking, so definitions of problematic use are underdeveloped, and risks and benefits of GenAI are not well understood. Regardless, our results suggest that intensive use of GenAI may, in a manner similar to general internet use, be more common among individuals at elevated risk for psychosis. Future research could address whether these relationships reflect a tendency of individuals with symptoms of psychosis to use AI, for AI use to influence symptoms, a third factor to influence both AI use and psychosis symptoms, or some combination of all these possibilities.

Second and relatedly, psychosis risk symptoms were associated with all measured motivations for using GenAI (likely reflecting a higher interest in chatbots among those at elevated risk); yet, this relationship was strongest for motivation to use GenAI for social and emotional support. While our cross-sectional data cannot speak to directionality of these associations, this finding aligns with previous results suggesting that online activities may be compensatory for unsatisfying or insufficient real-world social interactions and support [33]. Consistent with our findings on GenAI motivations, we also found that individuals at risk for psychosis were significantly more likely to ascribe human relationships to GenAI platforms. Participants at elevated risk were much more likely to see a GenAI system as their therapist, friend, romantic partner, or companion, although they did not differ in their likelihood to see AI as a sexual partner. Importantly, one driver of compulsive internet use among individuals experiencing mental health concerns is loneliness [15,33,34]. Since such platforms are easy to anthropomorphize [35], they may be seen more readily as a way to satisfy social needs, but it is unclear whether they may lead to greater benefits or risks in the short and long terms. One concern, for example, is that they could—through reducing incentives for human interactions—exacerbate loneliness over the long-term [36]. The fact that social support is a prominent motivation for GenAI use highlights the importance of research to answer this question.

Finally, we developed a new measure of GenAI interactions involving delusion-like experiences and evaluated the association of this measure with psychosis risk symptoms, finding a significant medium correlation (*r*=0.40, *P<*.001) between these 2 factors. Of the items on this new scale, no item was endorsed by a proportion smaller than 10% of the at-risk sample. This included around a quarter of the sample reporting that they used GenAI to make sense of special messages, the true nature of the world, or persecutory actions taken by others. This finding suggests that individuals with psychosis risk symptoms do interact with GenAI about topics related to paranoia, grandiosity, or conspiracy thinking. On one hand, this is intuitive. Users are likely to engage with GenAI systems about topics to which they dedicate thought. For individuals at risk for psychosis, this may include persecutory or grandiose ideas. However, given large-scale interactions with GenAI, the nature of responses provided by these systems are particularly important. If GenAI chatbots respond in sycophantic or reinforcing ways or provide misinformation, this could increase risks. If they respond in ways that reduce stigma and increase the likelihood of help-seeking, this could lead to benefits. Our data do not suggest that these delusion-related interactions are leading to either harm or benefit—such a claim would require prospective data; however, our results do indicate that interactions about symptom-related content appear to be happening on a scale that could lead to significant impact. Protective measures could reduce these risks or even leverage GenAI in a manner that could improve health outcomes, for example, by gently challenging problematic beliefs, destigmatizing help-seeking, or encouraging individuals to seek out treatment from a licensed professional.

**Limitations**

Our study is limited in several ways. First, these are cross-sectional data. Our analyses identify associations between variables where directionality remains unclear. It is possible that relationships reveal the influence psychosis risk has on GenAI use, vice versa, or some combination of the two. Future studies with longitudinal designs are necessary to answer questions about directionality. Second, data were collected via Prolific. Prolific users may not sufficiently represent the broader young adult population in the United States; a more inclusive or varied sampling strategy may do so more effectively. In particular, given their participation through an online crowdsourcing platform, our sample may be more likely to have used a variety of online tools, including GenAI, than the general population. Further, our sampling strategy involved collecting data from all Prolific users who responded to our study listing; a more intentional sampling strategy could have resulted in more generalizable results. Relatedly, while the PQ-B is a widely used validated assessment of prodromal symptoms, as mentioned, a high score on this scale on its own does not indicate that an individual meets clinical high-risk criteria. This determination is best made by a trained clinician. Third, given the novelty of this research area, many of the measures administered were developed for the proposed project. There also may be some conceptual overlap between measures of GenAI interactions and those that assess psychological constructs. While we based development of each measure on published literature and assessed psychometric attributes, future studies may reveal more comprehensive or robust measures. As is always the case with survey studies dependent on participant report, results may be impacted by response biases, or social desirability effects. Future designs should combine self-reported motivations for GenAI use with objective or passively collected device data.

### Conclusions

These findings speak to the importance of intentional and transparent design of GenAI systems to reduce risks to individuals at risk for psychosis. Individuals at elevated risk for psychosis appear more likely to use these tools with high frequency, to do so for social and emotional support, and to ascribe intimate relationships to chatbots. Given the intensity and frequency of these interactions, individuals at risk may be more sensitive to problematic content. Longitudinal research is needed to determine the real-world impact of GenAI on psychosis risk symptoms, but our study indicates that whatever their consequences, meaningful symptom-related interactions may be common among this population.

## Appendix

Multimedia Appendix 1 Item list for novel measures.

Multimedia Appendix 2 Supplemental analyses.

## Abbreviations

AI: artificial intelligence
AIMUS: Artificial Intelligence Motivation and Uses Scale
GAATES: Generative AI Aberrant Thoughts and Experiences Scale
GenAI: generative artificial intelligence
OR: odds ratio
PQ-B: Prodromal Questionnaire, Brief Version

## Data Availability

The datasets generated or analyzed during this study are available from the corresponding author on reasonable request.
