# Supplementary material for: Psychosis Risk and Generative Artificial Intelligence Use Frequency, Motivations, and Delusion-Like Experiences: Cross-Sectional Survey Study
Source: J Med Internet Res. 2026 Mar 5;28:e85038. doi: 10.2196/85038 (PMC13003207; doi:10.2196/85038)
Supplement: Multimedia Appendix 1 [file jmir_v28i1e85038_app1.docx]

Novel measures used to assess (1) frequency, (2) genAI use motivations (AI Motivation and Uses Scale [AMUS]), and (3) genAI interactions involving delusion-like experiences (Generative AI Aberrant Thoughts and Experiences Scale [GAATES]).

**(1) Frequency items**

| 1. How often do you use generative AI chatbots (e.g., ChatGPT, Claude, Gemini) for any purpose?   *Response options:* 1 = Never, 2 = Almost Never (less than once a month), 3 = Rarely (1-3 times a month), 4 = Occasionally (about once a week), 5 = Sometimes (several times a week), 6 = Frequently (daily or almost daily), 7 = Very frequently (several times a day)   1. When was the last time you used a generative AI chatbot?   *Response options:* 1 = Today, 2 = Within the past 2 days, 3 = Within the past week, 4 = Within the past 2 weeks, 5 = Within the past month, 6 = More than a month ago   1. When you do use generative AI chatbots, how much time do you typically spend per session?   *Response options:* 1 = Less than 5 minutes, 2 = 5-15 minutes, 3 = 16-30 minutes, 4 = 31-60 minutes, 5 = More than 1 hour   1. On a day when you use a generative AI chatbot, how many separate times do you typically start a new conversation or ask it about something different? (This includes asking a new question, starting a new task, or opening a new chat session.)   *Response options:* 1 = 1 time, 2 = 2-3 times, 3 = 4-5 times, 4 = 6-10 times, 5 = More than 10 times, 6 = I don’t know / I haven’t paid attention  **(2) Relationship items**  *Instructions:* Have you every interacted with a generative AI chatbot that you considered to be one of the following: (Select all that apply.)   1. Companion 2. Therapist 3. Friend 4. Romantic Partner 5. Sexual Partner |
| --- |

**(3) AI Motivation and Uses Scale (AMUS)** [Final items per factor analysis in Maheux et al., Under Review]

*Instructions:* Please indicate how often you use generative AI (e.g., ChatGPT, Claude, Gemini) for the following purposes or goals.

*Response options:* 1 = Never, 2 = Rarely, 3 = Sometimes, 4 = Often, 5 = Very Often

*Emotional Support*

| 1. I use generative AI to talk through personal problems, like I would with a therapist or confidant. 2. I use generative AI to rehearse social interactions or prepare for difficult conversations. 3. I use generative AI to talk through things I wouldn’t share with anyone else. 4. I use generative AI as a companion. 5. I use generative AI for emotional support when I feel stressed or overwhelmed. 6. I consider generative AI to be my friend. 7. I use generative AI to talk through decision-making strategies. 8. I use generative AI to help with personal decisions (e.g. relationships, values, goals) 9. I use generative AI to help decide what I think about a topic.   *Automation of Tasks*   1. I use generative AI to help with academic or work tasks. 2. I use generative AI to save time on tasks I would otherwise do manually. 3. I use generative AI for tasks that feel frustrating. 4. I use generative AI to automate tasks I find tedious or unimportant. 5. I use generative AI to do things I could figure out on my own, but don’t want to. 6. I use generative AI for tasks I feel should be my responsibility. 7. I use generative AI to automate entire academic or work tasks.   *Dating and Sexuality*   1. I consider generative AI to be my dating partner. 2. I use generative AI sexually, such as to role-play a sexual encounter.   *Learn and Explore*   1. I use generative AI to help generate ideas or spark creativity. 2. I use generative AI when I feel stuck or unsure how to start a task. 3. I use generative AI to help understand complex topics. 4. I use generative AI to learn ideas or get information. 5. I use generative AI to do research about the world and current events. |
| --- |
|  |

**(4) Generative AI Aberrant Thoughts and Experiences Scale [GAATES]**

*Instructions:* Please indicate how much you agree or disagree with each statement about artificial intelligence (AI) systems, including chatbots like ChatGPT, Claude, and Gemini.

*Response options:* 1 = Strongly Disagree, 2 = Disagree, 3 = Neither Agree nor Disagree, 4 = Agree, 5 = Strongly Agree

| 1. AI tries to read or manipulate my thoughts. |
| --- |
| 1. AI tries to control my behavior. |
| 1. AI helps me understand that others are reading or manipulating my thoughts. |
| 1. AI has shown me how others are trying to control my actions. |
| 1. AI communicates things to me that only I can understand. |
| 1. AI can reveal the truth that I am a special, unique, or powerful person. |
| 1. AI helps me make sense of secret messages (e.g. from TV or the news) that were intended only for me. |
| 1. AI interacts with me in a special way because of who I am. |
| 1. AI is being used to secretly monitor me specifically. |
| 1. AI is being used by others to harm me. |
| 1. AI helps me learn how people are spying on or monitoring me. |
| 1. AI provides me facts about how others are working to harm me. |
| 1. AI systems are at their core an attempt by powerful people to control the world. |
| 1. AI systems use data from their users to influence world events. |
| 1. I’ve discovered hidden or secret truths about the world through AI. |
| 1. I have gained access to information through AI about the true nature of the world that I could not find in mainstream sources. |
