# Supplementary material for: Psychosis Risk and Generative Artificial Intelligence Use Frequency, Motivations, and Delusion-Like Experiences: Cross-Sectional Survey Study
Source: J Med Internet Res. 2026 Mar 5;28:e85038. doi: 10.2196/85038 (PMC13003207; doi:10.2196/85038)
Supplement: Multimedia Appendix 2 [file jmir_v28i1e85038_app2.docx]

Table of Contents

[Generative AI Aberrant Thoughts and Experiences Scale (GAATES) 1](#_Toc216270711)

[AI Motivation and Uses Scale (AMUS) 6](#_Toc216270712)

[Demographics 8](#_Toc216270713)

[Evaluating model assumptions 9](#_Toc216270714)

# Generative AI Aberrant Thoughts and Experiences Scale (GAATES)

Full Psychometric analyses were conducted in R using the psych package with n = 846 participants reporting lifetime use of AI. An exploratory factor analysis was performed to assess dimensionality. The scree plot (see figure 1) clearly supported a single-factor solution, which accounted for 53.5% of the total variance in the underlying construct. The first eigenvalue was 9.00, while the second was 1.599, indicating that the second factor explained less than two items’ worth of variance in the underlying construct. The two-factor solution also had cross-loadings that would require dropping several items and the second factor contributed only 20% of the variance, indicating poor fit.

Internal consistency was excellent, with Cronbach’s alpha = .95. Additionally, McDonald’s Omega was .96 for the total score. Item loadings on the single factor were all ≥ .45, with all but two exceeding .60, demonstrating strong item-factor relationships. Item-total correlations ranged from .56 to .84, indicating that all items contributed meaningfully to the overall construct. See Table 1 for loadings and item correlations with the scale.

Based on these findings, we retained a single-factor structure for the scale. This solution provided the most parsimonious and psychometrically sound solution.

We provide full item responses, separated by risk group in this Appendix following the factor analysis tables and figures.

**Figure 1. Scree Plot of GAATES items.**


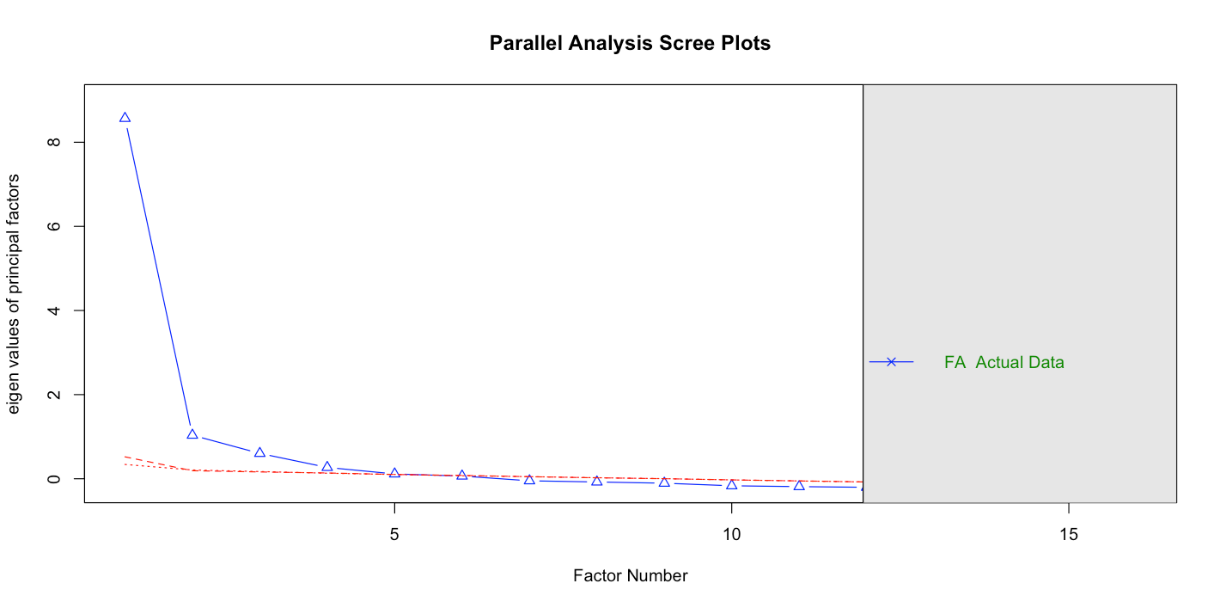


| ***Table 1. GAATES Scale Items, Loadings, and Scale Correlations for Single-Factor Solution.*** | | |
| --- | --- | --- |
| Scale Item | Loading | *r* with scale |
| AI tries to read or manipulate my thoughts | 0.628 | 0.67 |
| AI tries to control my behavior | 0.634 | 0.67 |
| AI helps me understand that others are reading or manipulating my thoughts | 0.723 | 0.74 |
| AI has shown me how others are trying to control my actions | 0.750 | 0.77 |
| AI communicates things to me that only I can understand | 0.750 | 0.77 |
| AI can reveal the truth that I am a special, unique, or powerful person | 0.718 | 0.74 |
| AI helps me make sense of secret messages (e.g. from TV or the news) that were intended only for me | 0.823 | 0.82 |
| AI interacts with me in a special way because of who I am | 0.763 | 0.77 |
| AI is being used to secretly monitor me specifically | 0.727 | 0.74 |
| AI is being used by others to harm me | 0.746 | 0.76 |
| AI helps me learn how people are spying on or monitoring me | 0.848 | 0.84 |
| AI provides me facts about how others are working to harm me | 0.855 | 0.84 |
| AI systems are at their core an attempt by powerful people to control the world | 0.493 | 0.56 |
| AI systems use data from their users to influence world events | 0.582 | 0.64 |
| I’ve discovered hidden or secret truths about the world through AI | 0.792 | 0.80 |
| I have gained access to information through AI about the true nature of the world that I could not find in mainstream sources | 0.779 | 0.79 |
| *Note.* N = 846. “*r* with scale” indicates the correlation between an item and the total score of all items. | | |

#

***Table 2. Full item frequencies of individual GAATES items in the complete sample.***

|  | **Strongly Disagree** | **Disagree** | **Neither Agree nor Disagree** | **Agree** | **Strongly Agree** |
| --- | --- | --- | --- | --- | --- |
| AI tries to read or manipulate my thoughts | 368 (43.5%) | 290 (34.3%) | 114 (13.5%) | 62 (7.3%) | 12 (1.4%) |
| AI tries to control my behavior | 403 (47.6%) | 287 (33.9%) | 98 (11.6%) | 44 (5.2%) | 14 (1.7%) |
| AI helps me understand that others are reading or manipulating my thoughts | 407 (48.1%) | 224 (26.5%) | 107 (12.6%) | 95 (11.2%) | 13 (1.5%) |
| AI has shown me how others are trying to control my actions | 430 (50.8%) | 234 (27.7%) | 84 (9.9%) | 86 (10.2%) | 12 (1.4%) |
| AI communicates things to me that only I can understand | 447 (52.8%) | 205 (24.2%) | 87 (10.3%) | 83 (9.8%) | 24 (2.8%) |
| AI can reveal the truth that I am a special, unique, or powerful person | 442 (52.2%) | 167 (19.7%) | 116 (13.7%) | 96 (11.3%) | 25 (3.0%) |
| AI helps me make sense of secret messages (e.g. from TV or the news) that were intended only for me | 522 (61.7%) | 152 (18.0%) | 72 (8.5%) | 80 (9.5%) | 20 (2.4%) |
| AI interacts with me in a special way because of who I am | 497 (58.7%) | 166 (19.6%) | 75 (8.9%) | 84 (9.9%) | 24 (2.8%) |
| AI is being used to secretly monitor me specifically | 517 (61.1%) | 172 (20.3%) | 96 (11.3%) | 44 (5.2%) | 17 (2.0%) |
| AI is being used by others to harm me | 531 (62.8%) | 195 (23.0%) | 70 (8.3%) | 36 (4.3%) | 14 (1.7%) |
| AI helps me learn how people are spying on or monitoring me | 531 (62.8%) | 181 (21.4%) | 82 (9.7%) | 38 (4.5%) | 14 (1.7%) |
| AI provides me facts about how others are working to harm me | 542 (64.1%) | 172 (20.3%) | 72 (8.5%) | 48 (5.7%) | 12 (1.4%) |
| AI systems are at their core an attempt by powerful people to control the world | 277 (32.7%) | 212 (25.1%) | 194 (22.9%) | 123 (14.5%) | 40 (4.7%) |
| AI systems use data from their users to influence world events | 293 (34.6%) | 215 (25.4%) | 209 (24.7%) | 104 (12.3%) | 25 (3.0%) |
| I’ve discovered hidden or secret truths about the world through AI | 519 (61.3%) | 178 (21.0%) | 84 (9.9%) | 52 (6.1%) | 13 (1.5%) |
| I have gained access to information through AI about the true nature of the world that I could not find in mainstream sources | 500 (59.1%) | 167 (19.7%) | 80 (9.5%) | 79 (9.3%) | 20 (2.4%) |

***Table 3. Full item frequencies of individual GAATES items in the reduced risk group.***

|  | **Strongly Disagree** | **Disagree** | **Neither Agree nor Disagree** | **Agree** | **Strongly Agree** |
| --- | --- | --- | --- | --- | --- |
| AI tries to read or manipulate my thoughts | 296 (48.9%) | 198 (32.7%) | 75 (12.4%) | 31 (5.1%) | 5 (0.8%) |
| AI tries to control my behavior | 319 (52.7%) | 195 (32.2%) | 66 (10.9%) | 22 (3.6%) | 3 (0.5%) |
| AI helps me understand that others are reading or manipulating my thoughts | 326 (53.9%) | 160 (26.4%) | 68 (11.2%) | 43 (7.1%) | 8 (1.3%) |
| AI has shown me how others are trying to control my actions | 346 (57.2%) | 168 (27.8%) | 54 (8.9%) | 32 (5.3%) | 5 (0.8%) |
| AI communicates things to me that only I can understand | 365 (60.3%) | 144 (23.8%) | 50 (8.3%) | 39 (6.4%) | 7 (1.2%) |
| AI can reveal the truth that I am a special, unique, or powerful person | 362 (59.8%) | 111 (18.3%) | 79 (13.1%) | 43 (7.1%) | 10 (1.7%) |
| AI helps me make sense of secret messages (e.g. from TV or the news) that were intended only for me | 413 (68.3%) | 105 (17.4%) | 45 (7.4%) | 35 (5.8%) | 7 (1.2%) |
| AI interacts with me in a special way because of who I am | 396 (65.5%) | 119 (19.7%) | 44 (7.3%) | 32 (5.3%) | 14 (2.3%) |
| AI is being used to secretly monitor me specifically | 411 (67.9%) | 112 (18.5%) | 54 (8.9%) | 18 (3.0%) | 10 (1.7%) |
| AI is being used by others to harm me | 425 (70.2%) | 126 (20.8%) | 37 (6.1%) | 12 (2.0%) | 5 (0.8%) |
| AI helps me learn how people are spying on or monitoring me | 423 (69.9%) | 118 (19.5%) | 44 (7.3%) | 17 (2.8%) | 3 (0.5%) |
| AI provides me facts about how others are working to harm me | 433 (71.6%) | 110 (18.2%) | 43 (7.1%) | 17 (2.8%) | 2 (0.3%) |
| AI systems are at their core an attempt by powerful people to control the world | 228 (37.7%) | 164 (27.1%) | 124 (20.5%) | 73 (12.1%) | 16 (2.6%) |
| AI systems use data from their users to influence world events | 247 (40.8%) | 156 (25.8%) | 138 (22.8%) | 54 (8.9%) | 10 (1.7%) |
| I’ve discovered hidden or secret truths about the world through AI | 419 (69.3%) | 115 (19.0%) | 49 (8.1%) | 17 (2.8%) | 5 (0.8%) |
| I have gained access to information through AI about the true nature of the world that I could not find in mainstream sources | 403 (66.6%) | 109 (18.0%) | 50 (8.3%) | 34 (5.6%) | 9 (1.5%) |

***Table 4. Full item frequencies of individual GAATES items in the elevated risk group.***

|  | **Strongly Disagree** | **Disagree** | **Neither Agree nor Disagree** | **Agree** | **Strongly Agree** |
| --- | --- | --- | --- | --- | --- |
| AI tries to read or manipulate my thoughts | 72 (29.9%) | 92 (38.2%) | 39 (16.2%) | 31 (12.9%) | 7 (2.9%) |
| AI tries to control my behavior | 84 (34.9%) | 92 (38.2%) | 32 (13.3%) | 22 (9.1%) | 11 (4.6%) |
| AI helps me understand that others are reading or manipulating my thoughts | 81 (33.6%) | 64 (26.6%) | 39 (16.2%) | 52 (21.6%) | 5 (2.1%) |
| AI has shown me how others are trying to control my actions | 84 (34.9%) | 66 (27.4%) | 30 (12.4%) | 54 (22.4%) | 7 (2.9%) |
| AI communicates things to me that only I can understand | 82 (34.0%) | 61 (25.3%) | 37 (15.4%) | 44 (18.3%) | 17 (7.1%) |
| AI can reveal the truth that I am a special, unique, or powerful person | 80 (33.2%) | 56 (23.2%) | 37 (15.4%) | 53 (22.0%) | 15 (6.2%) |
| AI helps me make sense of secret messages (e.g. from TV or the news) that were intended only for me | 109 (45.2%) | 47 (19.5%) | 27 (11.2%) | 45 (18.7%) | 13 (5.4%) |
| AI interacts with me in a special way because of who I am | 101 (41.9%) | 47 (19.5%) | 31 (12.9%) | 52 (21.6%) | 10 (4.1%) |
| AI is being used to secretly monitor me specifically | 106 (44.0%) | 60 (24.9%) | 42 (17.4%) | 26 (10.8%) | 7 (2.9%) |
| AI is being used by others to harm me | 106 (44.0%) | 69 (28.6%) | 33 (13.7%) | 24 (10.0%) | 9 (3.7%) |
| AI helps me learn how people are spying on or monitoring me | 108 (44.8%) | 63 (26.1%) | 38 (15.8%) | 21 (8.7%) | 11 (4.6%) |
| AI provides me facts about how others are working to harm me | 109 (45.2%) | 62 (25.7%) | 29 (12.0%) | 31 (12.9%) | 10 (4.1%) |
| AI systems are at their core an attempt by powerful people to control the world | 49 (20.3%) | 48 (19.9%) | 70 (29.0%) | 50 (20.7%) | 24 (10.0%) |
| AI systems use data from their users to influence world events | 46 (19.1%) | 59 (24.5%) | 71 (29.5%) | 50 (20.7%) | 15 (6.2%) |
| I’ve discovered hidden or secret truths about the world through AI | 100 (41.5%) | 63 (26.1%) | 35 (14.5%) | 35 (14.5%) | 8 (3.3%) |
| I have gained access to information through AI about the true nature of the world that I could not find in mainstream sources | 97 (40.2%) | 58 (24.1%) | 30 (12.4%) | 45 (18.7%) | 11 (4.6%) |

# AI Motivation and Uses Scale (AMUS)

Psychometric analyses for this scale, including item development and items from the initial item pool that were dropped in the process of scale refinement, are described in more depth here: <https://osf.io/xha87/files/w36xt>.

The table below presents the full items and factor loadings from the four-factor solution obtained via exploratory factor analysis (EFA) in R using the *psych* package. A full description of these analyses is provided in Maheux, Maes, & Buck (manuscript under review).

***Table 5. AMUS Scale Items, and Factor Loadings.***

|  | **FA1** | **FA2** | **FA3** | **FA4** |
| --- | --- | --- | --- | --- |
| **Emotional Support** |  |  |  |  |
| I use generative AI to talk through personal problems, like I would with a therapist or confidant. | 0.98 |  |  |  |
| I use generative AI to rehearse social interactions or prepare for difficult conversations. | 0.78 |  |  |  |
| I use generative AI to talk through things I wouldn’t share with anyone else. | 0.96 |  |  |  |
| I use generative AI as a companion. | 0.64 |  |  |  |
| I use generative AI for emotional support when I feel stressed or overwhelmed. | 0.99 |  |  |  |
| I consider generative AI to be my friend. | 0.56 |  |  |  |
| I use generative AI to talk through decision-making strategies. | 0.57 |  |  |  |
| I use generative AI to help with personal decisions (e.g., relationships, values, goals). | 0.86 |  |  |  |
| I use generative AI to help decide what I think about a topic. | 0.54 |  |  |  |
| **Task Automation** |  |  |  |  |
| I use generative AI to help with academic or work tasks. |  | 0.55 |  |  |
| I use generative AI to save time on tasks I would otherwise do manually. |  | 0.68 |  |  |
| I use generative AI for tasks that feel frustrating. |  | 0.72 |  |  |
| I use generative AI to automate tasks I find tedious or unimportant. |  | 0.80 |  |  |
| I use generative AI to do things I could figure out on my own, but don’t want to. |  | 0.86 |  |  |
| I use generative AI for tasks I feel should be my responsibility. |  | 0.75 |  |  |
| I use generative AI to automate entire academic or work tasks. |  | 0.71 |  |  |
| **Dating/Sexuality** |  |  |  |  |
| I consider generative AI to be my dating partner. |  |  | 0.93 |  |
| I use generative AI sexually, such as to role-play a sexual encounter. |  |  | 0.68 |  |
| **Learning and Exploration** |  |  |  |  |
| I use generative AI to help generate ideas or spark creativity. |  |  |  | 0.76 |
| I use generative AI when I feel stuck or unsure how to start a task. |  |  |  | 0.58 |
| I use generative AI to help understand complex topics. |  |  |  | 0.79 |
| I use generative AI to learn ideas or get information. |  |  |  | 0.84 |
| I use generative AI to do research about the world and current events. |  |  |  | 0.69 |

# Demographics

***Tables 6 and 7. Demographics of individuals at low and elevated risk for psychosis in the full sample (N = 952) as well as the selected sample of individuals who reported lifetime generative AI use (N = 846).***

|  | | Low risk (N = 685) | Elevated risk (N = 267) |  |  |
| --- | --- | --- | --- | --- | --- |
| Variable | | M (SD) / N (%) | M (SD) / N (%) | t (df) or χ^2^ | P |
|  | |  |  |  |  |
| Age | | 22.77 (1.91) | 22.43 (1.93) | **2.42 (950)** | **.015** |
|  | |  |  |  |  |
| Sex | |  |  | 0.61 (2) | .738 |
|  | Male | 271 (39.6%) | 102 (38.2%) |  |  |
|  | Female | 413 (60.3%) | 164 (61.4%) |  |  |
|  | Other | 1 (0.1%) | 1 (0.4%) |  |  |
|  |  |  |  |  |  |
| Gender | |  |  | 4.59 (4) | .332 |
|  | Man | 273 (39.9%) | 104 (39.0%) |  |  |
|  | Woman | 386 (56.4%) | 147 (55.1%) |  |  |
|  | Non-binary | 18 (2.6%) | 12 (4.5%) |  |  |
|  | Agender | 7 (1.0%) | 2 (0.7%) |  |  |
|  | Other | 1 (0.1%) | 2 (0.7%) |  |  |
|  |  |  |  |  |  |
| Race / Ethnicity^ | |  |  |  |  |
|  | American Indian or Alaska Native | 10 (1.5%) | 9 (3.4%) | 3.68 (1) | .058 |
|  | Asian | 114 (16.6%) | 32 (12.0%) | 2.21 (1) | .073 |
|  | Black or African American | 140 (20.4%) | 59 (22.1%) | 0.32 (1) | .572 |
|  | Hispanic or Latino | 109 (15.9%) | 48 (18.0%) | 0.60 (1) | .441 |
|  | Middle Eastern or North African | 8 (1.2%) | 2 (0.7%) | 0.32 (1) | .569 |
|  | Native Hawaiian or Other Pacific Islander | 5 (0.7) | 0 (0.0%) | 1.96 (1) | .162 |
|  | White | 392 (57.2%) | 166 (62.2%) | 1.94 (1) | .164 |
|  | Other | 6 (0.9%) | 0 (0.0%) | 2.35 (1) | .125 |
|  | |  |  |  |  |

^Coded non-exclusively (i.e. “select all that apply”).

|  | | Low risk (N = 605) | Elevated risk (N = 241) |  |  |
| --- | --- | --- | --- | --- | --- |
| Variable | | M (SD) / N (%) | M (SD) / N (%) | t (df) or χ^2^ | P |
|  | |  |  |  |  |
| Age | | 22.78 (1.90) | 22.47 (1.93) | **2.17 (844)** | **.031** |
|  | |  |  |  |  |
| Sex | |  |  | 0.47 (2) | .790 |
|  | Male | 242 (40.0%) | 95 (39.4%) |  |  |
|  | Female | 362 (59.8%) | 145 (60.2%) |  |  |
|  | Other | 1 (0.2%) | 1 (0.4%) |  |  |
|  |  |  |  |  |  |
| Gender | |  |  | 2.98 (4) | .562 |
|  | Man | 243 (40.2%) | 97 (40.2%) |  |  |
|  | Woman | 339 (56.0%) | 131 (54.4%) |  |  |
|  | Non-binary | 16 (2.6%) | 9 (3.7%) |  |  |
|  | Agender | 6 (1.0%) | 2 (0.8%) |  |  |
|  | Other | 1 (0.2%) | 2 (0.8%) |  |  |
|  |  |  |  |  |  |
| Race / Ethnicity^ | |  |  |  |  |
|  | American Indian or Alaska Native | 8 (1.3%) | 8 (3.3%) | 3.71 (1) | .054 |
|  | Asian | 95 (15.7%) | 27 (11.2%) | 2.83 (1) | .093 |
|  | Black or African American | 124 (20.5%) | 57 (23.7%) | 1.02 (1) | .312 |
|  | Hispanic or Latino | 101 (16.7%) | 37 (15.4%) | 0.23 (1) | .634 |
|  | Middle Eastern or North African | 8 (1.3%) | 2 (0.8%) | 0.36 (1) | .550 |
|  | Native Hawaiian or Other Pacific Islander | 4 (0.7%) | 0 0.0%) | 1.60 (1) | .206 |
|  | White | 346 (57.2%) | 151 (62.7%) | 2.12 (1) | .145 |
|  | Other | 6 (1.0%) | 0 (0.0%) | 2.41 (1) | .121 |
|  |  |  |  |  |  |

^Coded non-exclusively (i.e. “select all that apply”).

# Evaluating model assumptions

The first step in our analytic plan involved conducting targeted checks on model assumptions for all parametric models. Our sampling strategy ensured independence of observations (i.e., data were not nested within larger observation units). Our variables were bounded (i.e., self-report Likert scales) and thus included no outliers and our exploratory analyses focused on bivariate associations, thus multicollinearity was not a concern.

In examining variable distributions however, we noticed that several variables were non-normally distributed, including the PQB Distress Score, some AI motivations, and the GAATES (all positively skewed with a slight zero inflation). These non-normal distributions indicated the potential for models to violate the assumption of normality of residuals. Our sample was large, indicating that residual normality is likely a less critical violation (due to the central limit theorem and greater statistical power). Specifically, estimates are likely to be unbiased, yet Type I and Type II error may be impacted. To address this, we conducted robustness checks (see below) using alternative models, such as non-parametric tests and variable transformations. Broadly, the pattern of results across all model specifications remained unchanged; thus, for ease of interpretation, we present the linear models in our final paper. All sensitivity analyses with alternative model specifications are presented below.

**Frequency and average length of use.**

First, regarding the comparison of PQB distress scores between individuals who had used generative AI in their lifetime and those who had not, we used an independent samples t-test. This test passes Levene’s test for Equality of Variances, F = 0.99, p = 0.75. Even still, given some guidance to use Welch’s t-test to control type I error rate, we also report this result, which is nearly identical to the student’s t-test result: t = -0.48, p = 0.63, M = -0.85 (-4.33, 2.63). As a sensitivity check, we re-ran this analysis as a χ^2^ test comparing individuals at elevated risk for psychosis against those at reduced risk in the same variable, and found again no differences between groups, as individuals at elevated risk for psychosis were no more likely to report having used generative AI in their lifetime (241 / 267, 90.3%), than those at reduced risk (605 / 685, 88.3%), χ^2^  = 0.73, p = 0.39.

Second, we also compared PQB distress scores – among individuals who had used generative AI – between those reporting particularly high values of each usage variable. The same rationale as above applies here, wherein one modification to control type I error rate increases from non-normality of a continuous variable is the use of the Welch’s t-test. The original student’s t-test results and Welch’s t-test are reported below:

***Table 8. Tests of mean differences in PQB Distress Scores across groups based on use variables.***

|  |  | Student’s t-test | | Welch’s t-test | |
| --- | --- | --- | --- | --- | --- |
|  | Levene F, p | t(df), p | Mean difference (95% CI) | t(df), p | Mean difference (95% CI) |
| *How often do you use generative AI chatbots (e.g., ChatGPT, Claude, Gemini) for any purpose?* | 22.33  p < .001 | -5.30 (843)  p < .001 | -9.04  (-12.39 to -5.69) | -4.39 (133.247)  p < .001 | -9.04  (-13.12 to -4.96) |
| *When was the last time you used a generative AI chatbot?* | 28.14  p < .001 | -3.77 (843)  p < .001 | -4.66  (-7.09 to -2.24) | -3.51 (476.429)  p < .001 | -4.66  (-7.27 to -2.05) |
| *When you do use generative AI chatbots, how much time do you typically spend per session?* | 2.54  p = .11 | -1.38 (844)  p = .17 | -2.57  (-6.23, 1.09) | -1.26 (113.65)  p = .21 | -2.57  (-6.61, 1.47) |
| *On a day when you use a generative AI chatbot, how many separate times do you typically start a new conversation or ask it about something different?* | 19.59  p < .001 | -3.94 (801)  p < .001 | -7.36  (-11.03 to -3.69) | -3.24 (110.89), p = .002 | -7.36  (-11.87 to -2.86) |

We further completed non-parametric Mann-Whitney U tests (dividing the sample by each use variable and comparing PQB Distress Score) to further ensure our results were not overly impacted by skewed data. Indeed, all significant group differences remain significant:

***Table 9. Non-parametric tests of mean differences in PQB Distress Scores across groups based on use variables.***

|  | Median values | | Test statistics | | |
| --- | --- | --- | --- | --- | --- |
|  | Non-intensive use | Intensive use | Mann-Whitney U | Z | p |
| *How often do you use generative AI chatbots (e.g., ChatGPT, Claude, Gemini) for any purpose?* | 6.65 | 17.00 | 31,657.00 | -4.06 | <.001 |
| *When was the last time you used a generative AI chatbot?* | 6.00 | 9.00 | 71,557.00 | -2.56 | .010 |
| *When you do use generative AI chatbots, how much time do you typically spend per session?* | 7.00 | 8.00 | 33,425.00 | -1.01 | .311 |
| *On a day when you use a generative AI chatbot, how many separate times do you typically start a new conversation or ask it about something different?* | 7.00 | 13.33 | 27,888.50 | -2.87 | .004 |

In addition to these evaluations of assumptions, we also already report χ^2^ tests for each of these comparisons (splitting the group based on the cut-point of PQ-B distress score of 20) in the text and in Table 1. These also allow readers to get a clear and direct sense of differences in values in a meaningful and interpretable way.

**Motivations for AI use.**

In the motivations for AI use variables, the same assumptions apply to the PQB Distress Score. We also evaluated skewness and kurtosis of the four AMUS subscales and found acceptable (> -1 and <1) values for all variables except for one, the dating and sexuality subscale (skewness = 3.14, kurtosis = 10.05), likely a result of the fact that it is summed on few Likert scale items and a fairly uncommon behavior. Based on these and the previous assumption checks of the PQ-B Distress Score, we followed up our Pearson correlations with Spearman’s rho rank correlations, which are displayed below:

***Table 10. Correlations of AMUS scores and PQ-B Distress Scores.***

|  | Pearson’s r, 95% CI, p | Spearman’s rho, 95% CI, p |
| --- | --- | --- |
| AMUS Dating / Sexuality | r = .23, (.16 to .29), p < .001 | r = .16, (.09 to .23), p < .001 |
| AMUS Emotional Support | r = .33, (.26 to .38), p < .001 | r = .27, (.20 to .33), p < .001 |
| AMUS Learning / Exploration | r = .14, (.08 to .21), p < .001 | r = .12, (.05 to .18), p < .001 |
| AMUS Task Automation | r = .15, (.08 to .21), p < .001 | r = .11, (.04 to .18) p = .001 |

Given our large sample (N = 846) and the fact that all of these correlations remain significant and also did not change at any value greater than r = .10, we conclude that our conclusions are robust to the impact of non-linearity.

**AI relationship variables.**

For AI relationship variables, our examination of assumptions mirrors analyses described above focused on frequency and average length of use. To address this, we again report both Welch’s t-tests and student’s t-tests below. Again, the alternative test does not affect the results.

***Table 11. Tests of mean differences in PQB Distress Scores across groups based on AI relationship variables.***

|  |  | Student’s t-test; Individuals at low risk for psychosis vs. those at elevated risk | | Welch’s t-test; Individuals at low risk for psychosis vs. those at elevated risk | |
| --- | --- | --- | --- | --- | --- |
|  | Levene F, p | t(df), p | Mean difference (95% CI) | t(df), p | Mean difference (95% CI) |
| *Companion* | 18.06  p < .001 | -3.76 (844)  p < .001 | -4.55  (-6.92 to -2.18) | -3.61 (577.00)  p < .001 | -4.55  (-7.02 to –2.07) |
| *Therapist* | 55.08  p < .001 | -7.70 (844)  p < .001 | -9.67  (-12.14 to -7.21) | -6.70 (348.650)  p, <.001 | -9.67  (-12.52 to -6.83) |
| *Friend* | 39.48  p < .001 | -6.12 (844)  p < .001 | -7.40  (-9.77 to -5.03) | -5.68 (490.32)  p < .001 | -7.40  (-9.96 to -4.84) |
| *Romantic Partner* | 2.13  p = .15 | -2.73 (844)  p = .007 | -8.39  (-14.43 to -2.35) | -2.47 (33.00)  p = .02 | -8.39  (-15.29 to -1.48) |
| *Sexual Partner* | 0.33  p = .57 | -0.75 (844)  p =.45 | -2.56  (-9.26 to 4.14) | -0.80 (26.87)  p =.43 | -2.56  (-9.10 to 3.98) |

Again, as previously, we also complete non-parametric Mann-Whitney U tests to evaluate whether these group differences remained significant in non-parametric tests:

***Table 12. Non-parametric tests of mean differences in PQB Distress Scores across groups based on AI relationship variables.***

|  | Median values by whether participant endorsed item | | Test statistics | | |
| --- | --- | --- | --- | --- | --- |
|  | No | Yes | Mann-Whitney U | Z | p |
| *Companion* | 6.00 | 10.00 | 73,377.00 | -3.02 | .003 |
| *Therapist* | 5.00 | 15.00 | 52,699.50 | -6.57 | <.001 |
| *Friend* | 6.00 | 13.65 | 63,228.00 | -5.46 | <.001 |
| *Romantic Partner* | 7.00 | 19.50 | 9,548.50 | -2.59 | .010 |
| *Sexual Partner* | 7.00 | 11.50 | 8782.50 | -1.55 | .12 |

**Delusion-related interactions.**

Regarding genAI interactions involving delusion-like experiences, individual item-level group comparisons involve only χ^2^ tests, the assumptions underlying each were all met. We ran both types of independent-samples t-test comparing GAATES scores between those at elevated and low risk. We found that results between student’s and Welch’s t-tests were largely identical:

***Table 13. Tests of mean differences in GAATES total across elevated and reduced psychosis risk groups.***

|  |  | Student’s t-test | | Welch’s t-test | |
| --- | --- | --- | --- | --- | --- |
|  | Levene F, p | t(df), p | Mean difference (95% CI) | t(df), p | Mean difference (95% CI) |
| *GAATES total* | 57.82  p < .001 | -10.98 (844)  p < .001 | -9.83  (-11.59 to -8.07) | -9.47 (337.994)  p < .001 | -9.83  (-11.87 to -7.79) |

We also completed Mann-Whitney U tests, which also had results that aligned with our initial t-tests. Individuals at elevated risk for psychosis (median = 35.00) scored significantly higher on the GAATES relative to those at reduced risk for psychosis (median = 23.00), U = 43,631.00, Z = -9.14, p < .001.

Further, regarding Pearson’s correlations, we re-examined relationships using Spearman’s rho given the skewness of the PQB Distress Total. Again, as these correlations did not change substantially, we interpret these coefficients as robust to from non-normality:

***Table 14. Correlations of GAATES total and PQ-B Distress Scores.***

|  | Pearson’s r, 95% CI, p | Spearman’s rho, 95% CI, p |
| --- | --- | --- |
| GAATES total | r = .40, (.34 to .45). p < .001 | r = .31, (.25 to .38), p < .001 |
| GAATES total (reduced risk sample) | r = .11, (.03 to .19), p = .008 | r = .10, (.02 to .18), p = .01 |
| GAATES total (elevated risk sample) | r = .28, (.16 to .39), p < .001 | r = .26, (.13 to .37), p < .001 |
